# An Effective Approach for Reliability-Based Sensitivity Analysis with the Principle of Maximum Entropy and Fractional Moments

**DOI:** 10.3390/e21070649

**Published:** 2019-07-01

**Authors:** Xufang Zhang, Jiankai Liu, Ying Yan, Mahesh Pandey

**Affiliations:** 1 School of Mechanical Engineering & Automation, Northeastern University, Shenyang 110819, China; 2 The Technology Center, Taiyuan Heavy Industry Co. Ltd., Taiyuan 030024, China; 3 Department of Civil & Environmental Engineering, University of Waterloo, Waterloo, ON N2L 3G1, Canada

**Keywords:** multiplicative dimensional reduction method, reliability-based sensitivity analysis, fractional moments, the principle of maximum entropy

## Abstract

The reliability-based sensitivity analysis requires to recursively evaluate a multivariate structural model for many failure probability levels. This is in general a computationally intensive task due to irregular integrations used to define the structural failure probability. In this regard, the performance function is first approximated by using the multiplicative dimensional reduction method in this paper, and an approximation for the reliability-based sensitivity index is derived based on the principle of maximum entropy and the fractional moment. Three examples in the literature are presented to examine the performance of this entropy-based approach against the brute-force Monte-Carlo simulation method. Results have shown that the multiplicative dimensional reduction based entropy approach is rather efficient and able to provide reliability estimation results for the reliability-based sensitivity analysis of a multivariate structural model.

## 1. Introduction

In recent years, advanced computational technologies allow to develop detailed simulation models for virtual analysis and design optimization of structural systems. A key issue in this respect is to identify significant parameters while considering inherent uncertainties associated with the geometry, the material property, and the structural load variables. A recognized way to account for the input uncertainty is resorting to the probability theory. This includes the use of the probability theory to quantify input random variables and the effective algorithm for uncertainty quantification of the multivariate stochastic model [1,2]. The reliability-based sensitivity analysis that evaluates the significance input random variables with respect to the structural failure probability has received considerable attentions [3,4]. Due to computationally demanding cost for the reliability-based sensitivity analysis with a rather small failure probability, numerical evaluation of the sensitivity index becomes a challenging task. To this end, the paper presents an effective approach for the reliability-based sensitivity analysis based on the principle of maximum entropy (MaxEnt) and the fractional moment.

An accurate estimation for the structural failure probability is a precondition for the reliability-based sensitivity analysis. In engineering realities, the structural failure probability is usually defined based on a multivariate performance function g(X), i.e., $P_{F}=\mathrm{Pr}\left[g\left(X\right)\leq 0\right]$. Herein, the input random vector $X={\left[X_{1},\cdots ,X_{n}\right]}^{T}$ consists of all input random variables, whereas the failure domain is defined as {∀x:g(x)≤0}. Particulary, a numerical transformation is necessary to determine statistically independent random variables [5]. Note that the reliability-based sensitivity index is mathematically defined as the partial derivative of $P_{F}$ with respect to the mean and the standard deviation of input random variables, i.e., $\partial P_{F}/\partial \mu_{i}$ and $\partial P_{F}/\partial \sigma_{i}$ (as i=1,⋯,n). Therefore, a positively defined sensitivity index implies an increase of the distribution parameter will determine an increased structural failure probability, whereas a negative valued sensitivity index implies an inverse controlling effect of the distribution parameter on the structural failure probability. Note that the sensitivity index for the standard deviation is always negative, and an increase of variability of input random variables will generally increase the variation of a structural response, which further increases the exceeding probability of the model response quantity with respect to a predefined response threshold as shown in numerical examples.

Numerical evaluation of the reliability-based sensitivity index depends largely on an accurate estimation of the structural failure probability. In this regard, the first/second-order reliability method was developed in the literature for an effective estimation of the structural reliability index [6,7,8]. In addition, Bucher and Bourgund [9] proposed to approximate g(X) with a regression model to deal with implicit performance functions. Similar techniques, e.g., the polynomial chaos expansion [10], the Kriging approximation [11], and the artificial neutral network, etc. were reported in the literature [12,13]. Once a surrogate model of the structural performance function is analytically or numerically available, the subsequent reliability and reliability-based sensitivity analysis can be alternatively realized by the brute-force Monte-Carlo simulation and the response surface model. However, if the structural reliability result is gradually varied during the design optimization process, one has to develop new surrogate models for the updated structural reliability result [14]. This motives the entropy-based approach for the reliability-based sensitivity analysis in this paper.

The reliability-based sensitivity index has been widely used to rank the significance of input random variables. Specially, the variance-based global sensitivity method was investigated in many literatures [15,16]. Based on the variance decomposition of a generalized multivariate structural model, it is possible to express the total output response variance as a combination of variance components that are related to each group of input random variables and their combinations. Instead, the reliability-based sensitivity index pays major attention on the relation between distribution parameters and the structural failure probability. In this respect, the application of the variance-based sensitivity result will be rather limited, if the response distribution function of a structural model is highly skewed [17].

To effectively realize the reliability-based sensitivity analysis, Guo and Du [18] proposed to use the FORM-based approach that is based on a linear approximation of the performance function at the most probable failure point. Song and Lu [19] investigated the subset simulation and the variance reduction technique for the probabilistic sensitivity analysis [20]. Since the reliability-based sensitivity analysis is always limited to a predefined level of the structural failure probability, one way to determine the overall sensitivity result is to repeat the whole simulation procedure many times for a various realizations of the structural failure probability value, which is referred to as the distribution-based sensitivity analysis in the literature [21]. To this end, the paper presents an effective approach for the reliability-based sensitivity analysis based on the MaxEnt approach. The structural response distribution is first estimated by using the entropy optimization. Contrary to integer moments that are used in previous investigations, the fractional moment that is approximated by using the multiplicative dimensional reduction method is employed to derive probability distribution of a multivariate structural model for the sensitivity analysis.

To summarize, the objective of this paper is to present an entropy-based approach for reliability-based sensitivity analysis for a structural model function represented by using multivariate random variables. The principle of maximum entropy with fractional moment (ME-FM) is used to determine an accurate estimation result for the structural response distribution. The moment-based and the distribution- based sensitivity measures are derived to rank the significance of input random variables. Several examples in the literature are presented to demonstrate potential applications of this moment and the reliability-based sensitivity method.

The rest of the manuscript is organized as follows. Section 2 briefly summarizes probability measures that are used in this paper to rank the significance of an input random variable. With the multiplicative dimensional reduction method (M-DRM), sensitivity indicators based on the moment and the reliability results are derived in Section 3. Three examples in the literature are presented in Section 4 to examine the effectiveness of this approach against the brute-force Monte-Carlo simulation method, and conclusions are summarized in Section 5.

## 2. Measures for the Probabilistic Sensitivity Analysis

The section first summarizes the moment-based and the reliability-based measures that are used for the sensitivity analysis of a structural model with multivariate input random variables. The procedure based on the brute-force Monte-Carlo simulation approach is assumed to provide benchmark results for numerical validations.

### 2.1. The Moment-Based Sensitivity Measure

To begin with, the sensitivity coefficient for a distribution parameter with respect to an $\alpha^{\mathrm{th}}$-order moment of a multivariate structural model Y=η(x) is generally defined as

(1) $$\frac{\partial M_{Y}^{\alpha }}{\partial \theta_{i}}=\frac{\partial }{\partial \theta_{i}}\int_{X}{\left[\eta \left(x\right)\right]}^{\alpha }f_{X}\left(x\right)\;dx$$

Herein, $M_{Y}^{\alpha }$ denotes an αth-order fractional moment of the structural response quantity, whereas $\theta_{i}$ represents the distribution parameter, e.g., the mean or the standard deviation, of the input random variable $X_{i}$.

Introduce the kernel function for various types of the random variable in the Appendix A [22]:

(2) $$\kappa_{\theta_{i}}\left(x_{i}\right)=\frac{\partial \log[f_{i}\left(x_{i}\right)]}{\partial \theta_{i}}$$

The moment-based sensitivity index can be rewritten as [23]

(3) $$\frac{\partial M_{Y}^{\alpha }}{\partial \theta_{i}}=\int_{X}{\left[\eta \left(x\right)\right]}^{\alpha }\cdot \frac{\partial f_{X}\left(x\right)}{\partial \theta_{i}}\;dx=\int_{X}{\left[\eta \left(x\right)\right]}^{\alpha }\cdot \frac{\partial \log[f_{i}\left(x_{i}\right)]}{\partial \theta_{i}}\cdot f_{X}\left(x\right)dx$$

which is rewritten in a compact form as

(4) $$\frac{\partial M_{Y}^{\alpha }}{\partial \theta_{i}}=E\left\{{\left[\eta \left(X\right)\right]}^{\alpha }\cdot \kappa_{\theta_{i}}\left(X_{i}\right)\right\}$$

Specially, the brute-force MCS method determines the moment-based sensitivity coefficient as [24]

(5) $$\frac{\partial {\hat{M}}_{Y}^{\alpha }}{\partial \theta_{i}}=\frac{1}{N_{\mathrm{MCS}}}\sum_{k=1}^{N_{\mathrm{MCS}}}\left\{{\left[\eta \left(x^{\left(k\right)}\right)\right]}^{\alpha }\cdot \kappa_{\theta_{i}}\left(x_{i}^{\left(k\right)}\right)\right\}$$

where, the vector $x^{\left(k\right)}={\left[x_{1}^{\left(k\right)},\cdots ,x_{n}^{\left(k\right)}\right]}^{T}$ denotes a *k*th sample of X. The random simulation result will be used as the benchmark in numerical examples to verify numerical accuracy of the proposed approach.

### 2.2. The Reliability-Based Sensitivity Measure

The performance function for structural reliability analysis is usually defined as a multivariate structural model Y=η(X) and its threshold parameter $y_{c}$:

(6) $$g\left(X\right)=y_{c}-\eta \left(X\right)$$

Herein, the model response quantity can be the structural maximum stress, the deformation, and the fundamental natural frequency as shown in numerical examples. Therefore, the structural failure probability can be evaluated as [1]

(7) $$P_{F}\left(y_{c}\right)=\int_{y_{c}\leq \eta \left(x\right)}f_{X}\left(x\right)dx$$

The reliability-based sensitivity index is defined as the derivative of the structural failure probability $P_{F}$ with respect to the distribution parameter of input random variables [25]:

(8) $$\frac{\partial P_{F}\left(y_{c}\right)}{\partial \theta_{i}}=\frac{\partial }{\partial \theta_{i}}\int_{y_{c}\leq \eta \left(x\right)}f_{X}\left(x\right)dx$$

Herein, the distribution parameter $\theta_{i}$ can be the mean $\mu_{i}$ or the standard deviation $\sigma_{i}$ of an input random variable.

It is would be rather computationally demanding, if the finite difference method is used to evaluate the reliability-based sensitivity index [26]:

(9) $$\frac{\partial P_{F}\left(y_{c}\right)}{\partial \theta_{i}}\approx \frac{P_{F}\left(\theta_{i}+\Delta \theta_{i};y_{c}\right)-P_{F}\left(\theta_{i};y_{c}\right)}{\Delta \theta_{i}}$$

where, $\Delta \theta_{i}$ is a small quantity compared to its nominal value of the distribution parameter. In addition to the numerical stability problem, one has to evaluate 2n times of the structural reliability problem.

Alternatively, the reliability-based sensitivity analysis can be realized by the brute-force Monte-Carlo simulation method [27]:

(10) $$\frac{\partial P_{F}\left(y_{c}\right)}{\partial \theta_{i}}=E\left[I\left(X;y_{c}\right)\cdot \kappa_{\theta_{i}}\left(X_{i}\right)\right]$$

where, the indicating function is defined as $I(x;y_{c})=1$ if $y_{c}\leq \eta \left(x\right)$ and zero otherwise. Therefore, with $N_{\mathrm{mcs}}$ samples of the input random vector, the small quantity can be numerically estimated as

(11) $$\frac{\partial {\hat{P}}_{F}\left(y_{c}\right)}{\partial \theta_{i}}=\frac{1}{N_{\mathrm{mcs}}}\sum_{k=1}^{N_{\mathrm{mcs}}}\left\{I\left(x^{\left(k\right)};y_{c}\right)\cdot \frac{\partial \log[f_{i}\left(x_{i}^{\left(k\right)}\right)]}{\partial \theta_{i}}\right\}$$

Note that the approach is employed in this paper to provide benchmark results to check numerical accuracy of the MaxEnt approach for the reliability-based sensitivity analysis.

With the determined probability distribution function $f_{Y}\left(y\right)$ of the uncertain response quantity Y=η(X), the structural failure probability $P_{F}$ can be numerically estimated as

(12) $$P_{F}\left(y_{c}\right)=1-F_{Y}\left(y_{c}\right)$$

which further derives the reliability-based sensitivity index as

(13) $$\frac{\partial P_{F}\left(y_{c}\right)}{\partial \theta_{i}}=-\frac{\partial F_{Y}\left(y_{c}\right)}{\partial \theta_{i}}$$

Therefore, based on an effective estimation of the structural response distribution, the reliability-based sensitivity analysis can be alternatively realized based on ${\hat{F}}_{Y}\left(y\right)$ and the corresponding threshold value $y_{c}$. To this end, an entropy-based procedure for the reliability-based sensitivity analysis is presented as follows.

## 3. An Entropy-Based Approach for the Reliability-Based Sensitivity Analysis

The reliability-based sensitivity analysis needs an accurate estimation result for the structural response distribution, whereas the gradient-based and the random simulation-based approaches are computationally demanding in reality [28]. Alternatively, the multivariate structural response function is first approximated as the product of low-dimensional functions. One direct benefit of this approximation is able to calculate fraction moments of the structural response Y=η(X). Then, an effective approach to recover the distribution $F_{Y}\left(y\right)$ is derived based on the principle of maximum entropy (MaxEnt) and the fractional moment. To begin with, a brief summary on the multiplicative dimensional reduction method is presented as follows.

### 3.1. A Brief Summary on the Multiplicative Dimensional Reduction Method

The key idea of the multiplicative dimensional reduction method is to represent a multivariate performance function as the product of a series low-dimensional function with an increasing dimensions [29,30]. In this regard, the univariate approximation for a general response function η(x) is given as

(14) $$\eta \left(x\right)\approx {\left[\eta \left(c\right)\right]}^{1-n}\cdot \prod_{i=1}^{n}\eta \left(c_{1},\cdots ,c_{i-1},x_{i},c_{i+1},\cdots ,c_{n}\right)$$

Herein, the constant vector c is defined as $c={\left[c_{1},\cdots ,c_{n}\right]}^{T}$.

Besides the univariate approximation, a bivariate result can be used to improve the accuracy of the approximation to some extents [31]:

(15) $$\eta \left(x\right)\approx \frac{{\left[\eta \left(c\right)\right]}^{\frac{\left(n-1)(n-2\right)}{2}}\cdot \prod_{i=1}^{n-1}\prod_{j=i+1}^{n}\eta \left(c_{1},\cdots ,c_{i-1},x_{i},c_{i+1},\cdots ,c_{j-1},x_{j},c_{j+1},\cdots ,c_{n}\right)}{\prod_{i=1}^{n}{\left[\eta (c_{1},\cdots ,c_{i-1},x_{i},c_{i+1},\cdots ,c_{n})\right]}^{n-2}}$$

where, the bivariate component function $\eta (c_{1},\cdots ,c_{i-1},x_{i},c_{i+1},\cdots ,c_{j-1},x_{j},c_{j+1},\cdots ,c_{n})$ is defined for bivariate input random variables $X_{i}$ and $X_{j}$ (∀i,j=1,⋯,n and i≠j). More details on an *S*-variate approximation of the M-DRM approach, the readers are directed to the literature [32].

### 3.2. Fractional Moments and the MaxEnt Distribution

Similar to the integer moment, the fractional moment of a structural response quantity is defined as

(16) $$M_{Y}^{\alpha }=\int_{X}{\left[\eta \left(x\right)\right]}^{\alpha }f_{X}\left(x\right)dx$$

Herein, the moment exponent α is a real number.

Following the multivariate Gaussian quadrature method, numerical result for the fractional moment can be obtained as [33]

(17) $$M_{Y}^{\alpha }≃\sum_{i_{1}=1}^{N_{1}}w_{1}^{\left(i_{1}\right)}\sum_{i_{2}=1}^{N_{2}}w_{2}^{\left(i_{2}\right)}\cdots \sum_{i_{n}=1}^{N_{n}}w_{n}^{\left(i_{n}\right)}{\left[\eta \left(x_{1}^{\left(i_{1}\right)},x_{2}^{\left(i_{2}\right)},\cdots ,x_{n}^{\left(i_{n}\right)}\right)\right]}^{\alpha }$$

where, $w_{i}^{\left(i_{k}\right)}$ and $x_{i}^{\left(i_{k}\right)}$ represent an $i_{k}$th Gaussian weight and abscissa used uniquely for the input random variable $X_{i}$. Note that there are totally $\prod_{i=1}^{n}N_{i}$ combinations of the integration grid $\left\{x_{1}^{\left(i_{1}\right)},\cdots ,x_{n}^{\left(i_{n}\right)}\right\}$. The multivariate Gaussian-quadrature rule, hence, will be particularly expensive as the dimensional parameter n≥3. This motives a mathematical approach to approximate a multivariate mechanistic model η(·) as the product of univariate and/or bivariate component functions in this paper.

Once the fractional moment of a structural response quantity is numerically or analytically available, the principle of maximum entropy (MaxEnt) can be used to determine an estimation of the response distribution:

(18) $${\hat{f}}_{Y}\left(y\right)=\exp\left(-\sum_{k=0}^{m}\lambda_{k}y^{\alpha_{k}}\right)$$

in which,

$$\alpha_{0}=0,\;\mathrm{and}\;\lambda_{0}=\log\left[\int_{Y}\exp\left(-\sum_{k=1}^{m}\lambda_{k}y^{\alpha_{k}}\right)dy\right]$$

Herein, unknown parameters α and λ in the entropy distribution can be determined by the following optimization procedure [34]:

(19) $$\left\{\begin{array}{cc} \mathrm{Find}:\; & \lambda \;\mathrm{and}\;\alpha \\ \mathrm{Minimize}:\; & I\left(\lambda ,\alpha \right)=\log\left[\int_{Y}\exp\left(-\sum_{k=1}^{m}\lambda_{k}y^{\alpha_{k}}\right)dy\right]+\sum_{k=1}^{m}\lambda_{k}M_{Y}^{\alpha_{k}} \end{array}\right.$$

in which, $M_{Y}^{\alpha_{k}}$ is an $\alpha_{k}^{\mathrm{th}}$ order fractional moment, which can be efficiently calculated by using the M-DRM approach as follows. Note that the parameter *m* represents the total number of fractional moments that are used for an estimation of the unknown probability distribution with the MaxEnt approach. In numerical examples, the parameter m=3 is used to recover the distribution function.

Note that the fractional moment provides much more information for an inference of the unknown probability distribution than that of integer moments [35,36,37]. Therefore, the probability distribution determined by maximizing the entropy under the fractional moment constraints is given the most rational choice for $f_{Y}\left(y\right)$. With the multiplicative dimensional reduction method to efficiently calculate the fraction moment, results in numerical examples will demonstrate the effectiveness the fractional moment based MaxEnt approach in estimating the probability distribution of a general structural response function.

### 3.3. The Proposed M-DRM Approach for the Moment-Based Sensitivity Index

The estimation for probability distribution of a generic multivariate structural response model Y=η(X) depends largely on the availability of fractional moments $M_{Y}^{\alpha }$. Numerical evaluation of the multivariate moment integral can be realized by the standard Gaussian quadrature scheme in Equation (17), which is embeded the curse of the dimensionality problem for multivariate cases. In this regard, the univariate M-DRM approximation is used in this paper to derive an effective approximation for the fractional moment result as follows.

To implement, the univariate M-DRM approximation in Equation (14) is first used to approximate the structural model function η(·). This approximates the fractional moment result as

(20) $$M_{Y}^{\alpha }\approx \int_{X}{\left\{{\left[\eta \left(c\right)\right]}^{1-n}\cdot \prod_{i=1}^{n}\eta \left(x_{i},c_{-i}\right)\right\}}^{\alpha }f_{X}\left(x\right)dx$$

With independent input random variables, one has $f_{X}\left(x\right)=\prod_{i=1}^{n}f_{i}\left(x_{i}\right)$, and the M-DRM approximation for the fractional moment can be further rewritten as

(21) $$M_{Y}^{\alpha }\approx {\left[\eta \left(c\right)\right]}^{\alpha -\alpha n}\cdot \prod_{i=1}^{n}E_{i}\left\{{\left[\eta (X_{i},c_{-i})\right]}^{\alpha }\right\}$$

Combined with the Gaussian-quadrature scheme in the literature [33] to deal with the univariate integration, the one-dimensional integrations in the moment estimation procedure can be numerical realized as

(22) $$E_{i}\left\{{\left[\eta (X_{i},c_{-i})\right]}^{\alpha }\right\}≃\sum_{k=1}^{N_{k}}w_{i}^{\left(k\right)}\;{\left[\eta \left(x_{i}^{\left(k\right)},c_{-i}\right)\right]}^{\alpha }$$

where, $w_{i}^{\left(k\right)}$ and $x_{i}^{\left(k\right)}$ represent a *k*th Gauss-weight and abscissa of an *i*th input random variable, respectively.

Following the univariate M-DRM procedure, a result of the moment-based sensitivity index $\partial M_{Y}^{\alpha }/\partial \theta_{i}$ derived in Equation (4) can be further approximated as

(23) $$\begin{array}{cc} \frac{\partial M_{Y}^{\alpha }}{\partial \theta_{i}}= & \int_{X}{\left[\eta \left(x\right)\right]}^{\alpha }\cdot \kappa_{\theta_{i}}\left(x_{i}\right)\cdot f_{X}\left(x\right)dx\\ \approx & \int_{X}{\left[\eta^{1-n}\left(c\right)\cdot \prod_{j=1}^{n}\eta \left(x_{j},c_{-j}\right)\right]}^{\alpha }\cdot \kappa_{\theta_{i}}\left(x_{i}\right)\cdot f_{X}\left(x\right)dx\\ = & {\left[\eta \left(c\right)\right]}^{\alpha -\alpha n}\cdot E_{i}\left\{{\left[\eta (X_{i},c_{-i})\right]}^{\alpha }\cdot \kappa_{\theta_{i}}\left(x_{i}\right)\right\}\cdot \prod_{j=1,j\neq i}^{n}E_{j}\left\{{\left[\eta (X_{j},c_{-j})\right]}^{\alpha }\right\} \end{array}$$

Denote the univariate integration as follows:

(24) $$\left\{\begin{array}{c} \rho_{i}\left(\alpha \right)=E_{i}\left\{{\left[\eta \left(X_{i},c_{-i}\right)\right]}^{\alpha }\right\}\\ \beta_{i}\left(\alpha \right)=E_{i}\left\{{\left[\eta \left(X_{i},c_{-i}\right)\right]}^{\alpha }\cdot \kappa_{\theta_{i}}\left(X_{i}\right)\right\} \end{array}\right.$$

together with the corresponding Gaussian-quadrature based approximation results:

(25) $$\left\{\begin{array}{c} \rho_{i}\left(\alpha \right)\approx \sum_{k=1}^{N_{k}}\left\{w_{i}^{\left(k\right)}\cdot {\left[\eta \left(c_{1},\cdots ,c_{i-1},x_{i}^{\left(k\right)},c_{i+1},\cdots ,c_{n}\right)\right]}^{\alpha }\right\}\\ \beta_{i}\left(\alpha \right)\approx \sum_{k=1}^{N_{k}}\left\{w_{i}^{\left(k\right)}\cdot {\left[\eta \left(c_{1},\cdots ,c_{i-1},x_{i}^{\left(k\right)},c_{i+1},\cdots ,c_{n}\right)\right]}^{\alpha }\cdot \kappa_{\theta_{i}}\left(x_{i}^{\left(k\right)}\right)\right\} \end{array}\right.$$

This finally determines the M-DRM approximation for the moment-based sensitivity index in Equation (23) as

(26) $$\frac{\partial {\hat{M}}_{Y}^{\alpha }}{\partial \theta_{i}}={\left[\eta \left(c\right)\right]}^{\alpha -\alpha n}\cdot \prod_{j=1,j\neq i}^{n}\rho_{j}\left(\alpha \right)\cdot \beta_{i}\left(\alpha \right)$$

which implies the total number of functional evaluations (FEs) for the univariate M-DRM based moment sensitivity analysis will be

(27) $$FE_{\mathrm{uiv}}=1+\sum_{i=1}^{n}N_{i}$$

This number is much smaller than that of the standard tensor product rule in Equation (17), i.e., $\prod_{i=1}^{n}N_{i}$.

### 3.4. The Proposed Approach for the Reliability-Based Sensitivity Analysis

Analytical derivation for the response distribution function of a general multivariate structural model is seldom applicable in engineering realities. Therefore, with the M-DRM approximation result for fractional moments, an estimation of the unknown distribution $f_{Y}\left(y\right)$ is possibly determined by using the principle maximum entropy (MaxEnt) and the fractional moment, and the corresponding reliability-based sensitivity results are further derived to rank the significance of distribution parameters as follows.

To begin with, the MaxEnt approximation for the cumulative distribution function $F_{Y}\left(y\right)$ of the structural response model Y=η(X) is determined as

(28) $${\hat{F}}_{Y}\left(y\right)=\int_{0}^{y}{\hat{f}}_{Y}\left(z\right)dz=\int_{0}^{y}\exp\left(-\sum_{i=0}^{m}\lambda_{i}z^{\alpha_{i}}\right)dz$$

With a threshold parameter $y_{c}$ in defining the structural performance function in Equation (6), the structural failure probability can be determined as

(29) $$P_{F}\left(y_{c}\right)=1-{\hat{F}}_{Y}\left(y_{c}\right)=\int_{y_{c}}^{+\infty }\exp\left(-\sum_{i=0}^{m}\lambda_{i}y^{\alpha_{i}}\right)dy$$

Note that the MaxEnt parameters $\lambda ={\left[\lambda_{0},\lambda_{1},\cdots ,\lambda_{m}\right]}^{T}$ and $\alpha ={\left[\alpha_{1},\cdots ,\alpha_{m}\right]}^{T}$ were numerically optimized with the procedure in Equation (19).

Therefore, with the reliability estimation result in Equation (29), the reliability-based sensitivity index $\partial P_{F}\left(y_{c}\right)/\partial \theta_{k}$ is derived as

(30) $$\frac{\partial P_{F}\left(y_{c}\right)}{\partial \theta_{k}}=\frac{\partial }{\partial \theta_{k}}\int_{y_{c}}^{+\infty }\exp\left(-\lambda_{0}-\sum_{i=1}^{m}\lambda_{i}y^{\alpha_{i}}\right)dy$$

Considering the moment-based sensitivity index in Equation (23), the reliability-based sensitivity index can be further rewritten as [38]

(31) $$\frac{\partial P_{F}\left(y_{c}\right)}{\partial \theta_{k}}=\sum_{j=0}^{m}\frac{\partial P_{F}\left(y_{c}\right)}{\partial \lambda_{j}}\left(\sum_{i=1}^{m}\frac{\partial \lambda_{j}}{\partial M_{Y}^{\alpha_{i}}}\cdot \frac{\partial M_{Y}^{\alpha_{i}}}{\partial \theta_{i}}\right)$$

Herein, the sensitivity coefficient $\partial P_{F}/\partial \lambda_{j}$ is expressed as

(32) $$\frac{\partial P_{F}\left(y_{c}\right)}{\partial \lambda_{j}}=-\int_{y_{c}}^{+\infty }y^{\alpha_{j}}\exp\left(-\sum_{i=0}^{m}\lambda_{i}\;y^{\alpha_{i}}\right)dy$$

To determine the partial derivatives $\partial \lambda_{j}/\partial M_{Y}^{\alpha_{i}}$, we consider an $\alpha_{i}^{\mathrm{th}}$ order fractional moment:

(33) $$M_{Y}^{\alpha_{i}}=\int_{Y}y^{\alpha_{i}}\cdot \exp\left(-\lambda_{0}-\sum_{i=1}^{m}\lambda_{i}y^{\alpha_{i}}\right)dy$$

which has the following partial derivative result:

(34) $$\frac{\partial M_{Y}^{\alpha_{i}}}{\partial \lambda_{j}}=\frac{\partial }{\partial \lambda_{j}}\int_{Y}y^{\alpha_{i}}\exp\left(-\lambda_{0}-\sum_{i=1}^{m}\lambda_{i}y^{\alpha_{i}}\right)dy=-M_{Y}^{\alpha_{i}+\alpha_{j}}\;\left(i=1,\cdots ,m;j=0,\cdots ,m;\alpha_{0}=0\right)$$

which is expressed as an $\left(\alpha_{i}+\alpha_{j}\right)$ th-order fractional moment of the structural response quantity. Specially, the mean-value based partial derivative results for the Lagrange multiplier are given as

(35) $$\frac{\partial \mu_{Y}}{\partial \lambda_{j}}=\frac{\partial M_{Y}^{1}}{\partial \lambda_{j}}=-M_{Y}^{1+\alpha_{j}}\;\left(j=0,\cdots ,m;\alpha_{0}=0\right)$$

Therefore, one has the following squared matrix for partial derivatives $\partial M_{Y}^{\alpha_{i}}/\partial \lambda_{j}$:

(36) $${\left[\frac{\partial M_{Y}^{\alpha }}{\partial \lambda }\right]}_{\left(m+1)\times (m+1\right)}=\left[\begin{array}{cccc} \frac{\partial \mu_{Y}}{\partial \lambda_{0}} & \frac{\partial \mu_{Y}}{\partial \lambda_{1}} & \cdots & \frac{\partial \mu_{Y}}{\partial \lambda_{m}}\\ \frac{\partial M_{Y}^{\alpha_{1}}}{\partial \lambda_{0}} & \frac{\partial M_{Y}^{\alpha_{1}}}{\partial \lambda_{1}} & \cdots & \frac{\partial M_{Y}^{\alpha_{1}}}{\partial \lambda_{m}}\\ \vdots & \vdots & \cdots & \vdots \\ \frac{\partial M_{Y}^{\alpha_{m}}}{\partial \lambda_{0}} & \frac{\partial M_{Y}^{\alpha_{m}}}{\partial \lambda_{1}} & \cdots & \frac{\partial M_{Y}^{\alpha_{m}}}{\partial \lambda_{m}} \end{array}\right]=-\left[\begin{array}{cccc} \mu_{Y} & M_{Y}^{1+\alpha_{1}} & \cdots & M_{Y}^{1+\alpha_{m}}\\ M_{Y}^{\alpha_{1}} & M_{Y}^{2\alpha_{1}} & \cdots & M_{Y}^{\alpha_{1}+\alpha_{m}}\\ \vdots & \vdots & \cdots & \vdots \\ M_{Y}^{\alpha_{m}} & M_{Y}^{\alpha_{m}+\alpha_{1}} & \cdots & M_{Y}^{2\alpha_{m}} \end{array}\right]$$

Considering that each element $\frac{\partial \lambda_{j}}{\partial M_{Y}^{\alpha_{i}}}=-M_{Y}^{-(\alpha_{i}+\alpha_{j})}$ and $\frac{\partial \lambda_{j}}{\partial \mu_{Y}}=-M_{Y}^{-(1+\alpha_{j})}$ (as $i=1,\cdots ,m;j=0,\cdots ,m;\alpha_{0}=0$), the gradient matrix $\left[\partial \lambda /\partial M_{Y}^{\alpha }\right]$ can be finally obtained as

(37) $${\left[\frac{\partial \lambda }{\partial M_{Y}^{\alpha }}\right]}_{\left(m+1)\times (m+1\right)}=\left[\begin{array}{cccc} \frac{\partial \lambda_{0}}{\partial \mu_{Y}} & \frac{\partial \lambda_{0}}{\partial M_{Y}^{\alpha_{1}}} & \cdots & \frac{\partial \lambda_{0}}{\partial M_{Y}^{\alpha_{m}}}\\ \frac{\partial \lambda_{1}}{\partial \mu_{Y}} & \frac{\partial \lambda_{1}}{\partial M_{Y}^{\alpha_{1}}} & \cdots & \frac{\partial \lambda_{1}}{\partial M_{Y}^{\alpha_{m}}}\\ \vdots & \vdots & \cdots & \vdots \\ \frac{\partial \lambda_{m}}{\partial \mu_{Y}} & \frac{\partial \lambda_{m}}{\partial M_{Y}^{\alpha_{1}}} & \cdots & \frac{\partial \lambda_{m}}{\partial M_{Y}^{\alpha_{m}}} \end{array}\right]=-{\left[\begin{array}{cccc} \mu_{Y}^{-1} & M_{Y}^{-(1+\alpha_{1})} & \cdots & M_{Y}^{-(1+\alpha_{m})}\\ M_{Y}^{-\alpha_{1}} & M_{Y}^{-2\alpha_{1}} & \cdots & M_{Y}^{-(\alpha_{1}+\alpha_{m})}\\ \vdots & \vdots & \cdots & \vdots \\ M_{Y}^{-\alpha_{m}} & M_{Y}^{-(\alpha_{m}+\alpha_{1})} & \cdots & M_{Y}^{-2\alpha_{m}} \end{array}\right]}^{T}$$

To summarize, the proposed approach for the reliability-based sensitivity analysis includes: (a) the optimization for the MaxEnt distribution of the structural response quantity in Equation (18); (b) the M-DRM approximation for the moment-based sensitivity index in Equation (23); (c) the calculation of the gradient matrix for the Lagrange multiplier in Equation (37); and (d) the estimation of the reliability-based sensitivity index in Equation (31). Specially, the reliability-based sensitivity coefficient is evaluated for an arbitrary realization of the threshold parameter $y_{c}$. It is easy to determine the distribution-based sensitivity index based on one-round simulation of the structural model, instead of repeated evaluating the structural failure probability for various realizations of the parameter $y_{c}$. In this regard, the proposed approach is much superior than that of the FORM-based approach in the literature [39].

## 4. Numerical Examples

Engineering applications of the proposed approach for the moment-based and the reliability-based sensitivity analysis are illustrated by three examples in this section. Numerical examples presented in Section 4.1 and Section 4.2 are explicitly defined with respect to the input random variables, whereas the natural frequency function of a vehicle frame structure in Section 4.3 is defined as an implicit function of geometry and material random variables. Compared with benchmark results provided by the brute-force Monte-Carlo simulation method, the performance of this MaxEnt approach is examined as follows.

To rank the importance of each distribution parameter, the following sensitivity indices in the literature [40] are used in this paper:

(38) $$\left\{\begin{array}{cc} \text{Moment-based sensitivity index}:\;\; & \frac{\partial {\hat{M}}_{Y}^{\alpha }}{\partial \mu_{i}}=\frac{\partial M_{Y}^{\alpha }}{\partial \mu_{i}}\cdot \sigma_{i};\;\frac{\partial {\hat{M}}_{Y}^{\alpha }}{\partial \sigma_{i}}=\frac{\partial M_{Y}^{\alpha }}{\partial \sigma_{i}}\cdot \sigma_{i};\\ \text{Reliability-based sensitivity index}: & \frac{\partial {\hat{P}}_{F}}{\partial \mu_{i}}=\frac{\partial P_{F}}{\partial \mu_{i}}\cdot \sigma_{i};\;\frac{\partial {\hat{P}}_{F}}{\partial \sigma_{i}}=\frac{\partial P_{F}}{\partial \sigma_{i}}\cdot \sigma_{i}; \end{array}\right.$$

where, $\mu_{i}$ and $\sigma_{i}$ are the mean and the standard deviation of an *i*th input random variable, and $M_{Y}^{\alpha }$ denotes an αth order moment of the structural response function Y=η(X). Specially, results for the reliability-based sensitivity index are dimensionless by multiplying with the standard deviation of the input random variable, and only results for the mean-value based moment sensitivity indices (α=1), i.e., $\partial {\hat{\mu }}_{Y}/\partial \mu_{i}$ and $\partial {\hat{\mu }}_{Y}/\partial \sigma_{i}$ are presented in numerical examples for the sake of brevity.

### 4.1. Reliability-Based Sensitivity Analysis A Cantilever Tube Structure

**Figure 1:**
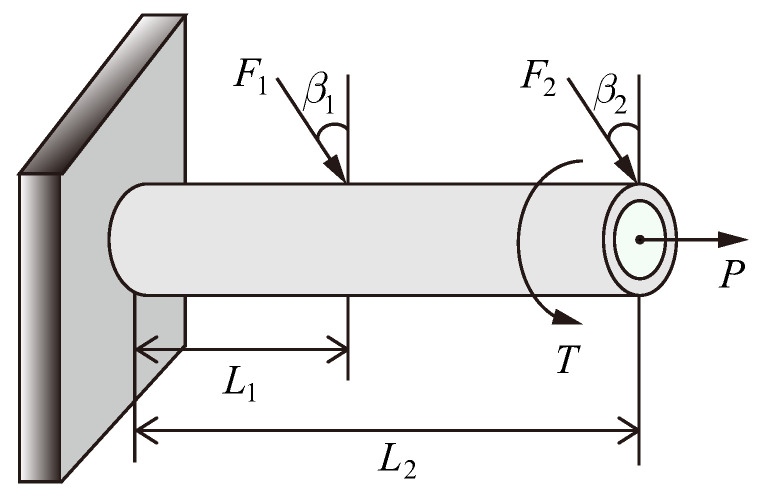
Example of a cantilever tube: $\beta_{1}=15^{\circ }$ and $\beta_{2}=25^{\circ }$.

This example considers the reliability-based sensitivity analysis of a cantilever tube structure depicted in Figure 1. The performance function is defined as

(39) $$g\left(X\right)=\frac{S_{y}}{\sigma_{\max}\left(X\right)}$$

where, $S_{y}$ denotes the material yield strength, and $\sigma_{\max}$ represents the structural maximum stress. Failure events of the cantilever tube structure are specified as $\left\{\forall x\in R^{n}:g\left(x\right)\leq 1.0\right\}$.

With external forces $F_{1}$, $F_{2}$, *P*, and *T*, the maximum von-Mises stress on the top surface of the tube is given as

(40) $$\sigma_{\max}=\sqrt{\sigma_{x}^{2}+2\tau_{zx}^{2}}$$

Herein, the torsion stress is defined as $\tau_{zx}=\frac{Td}{4I}$, whereas the normal stress $\sigma_{x}$ is given as

(41) $$\sigma_{x}=\frac{P+F_{1}\sin\left(\beta_{1}\right)+F_{2}\sin\left(\beta_{2}\right)}{A}+\frac{Mc}{I}$$

Specially, the parameters are given as $A=\frac{\pi }{4}\left[d^{2}+{\left(d-2t\right)}^{2}\right],c=d/2,I=\frac{\pi }{64}\left[d^{4}-{\left(d-2t\right)}^{4}\right]$. Therefore, the bending moment *M* is determined as

$$M=F_{1}L_{1}\cos\left(\beta_{1}\right)+F_{2}L_{2}\cos\left(\beta_{2}\right)$$

Note that *d* and *t* represent the outside diameter and the thickness of the tube, respectively. The probabilistic characteristic of input random variables are summarized in Table 1.

**Figure 2:**
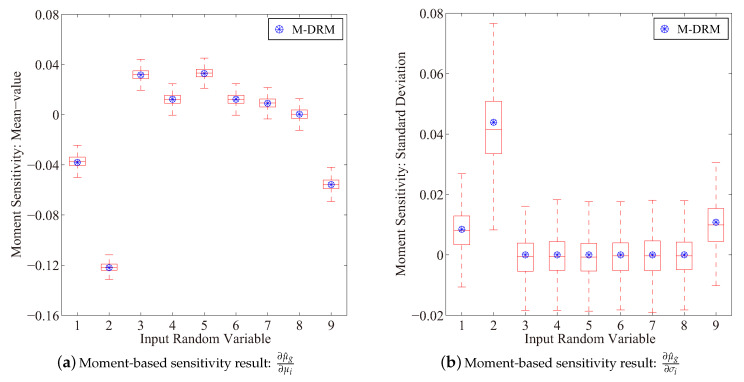
Results of the moment-based sensitivity index(Boxplot: 10^3^ rounds simulation with 10^3^ samples in each).

Figure 2 depicts the moment-based sensitivity result for the performance function in Equation (39). It is observed that distribution parameters $\mu_{1}$, $\mu_{2}$ and $\mu_{9}$ influence the performance function negatively, where other mean-value parameters, i.e., $\mu_{i}$ (i=3,⋯,8) have shown positive sensitivity results. Moreover, $X_{2}$ is identified as the most significant uncertain factor among all input variables to manipulate the mean-value response of the performance function, which is also justified by the reliability-based sensitivity result as follows.

**Figure 3:**
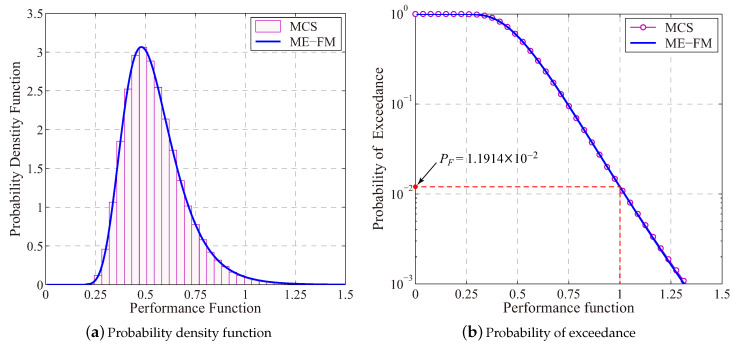
Results for the MaxEnt distribution of *g*(**X**) and the structural failure probability (The MCS approach with 10^5^ samples determines *P_F_* = 1.2035 × 10^−2^).

To derive probability distribution of structural performance function g(X), the procedure summarized in Equation (19) was implemented. Results for the MaxEnt parameter are summarized in Table 2, whereas Figure 3 presents simulation results for the probability distribution. With three-order fractional moments of the structural performance function i.e., m=3, an accurate result for the probability distribution of g(X) is determined as shown in Figure 3. With the determined result for the probability of exceedance, the structural failure probability is estimated as $P_{F}=1.1914\times 10^{-2}$, which is fairly close to the benchmark result $1.2035\times 10^{-2}$.

**Figure 4:**
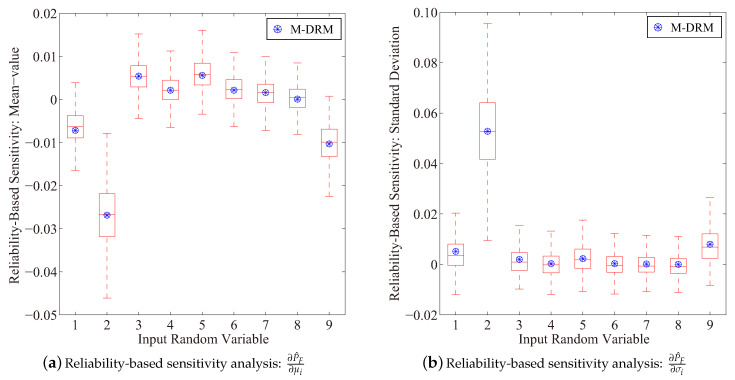
Results for the reliability-based sensitivity index of the cantilever tube example (Boxplot: 10^3^ rounds simulation with 10^3^ samples in each).

Figure 4 presents results for the reliability-based sensitivity analysis of the cantilever tube example. The simulation result is determined based on $10^{3}$ rounds brute-force Monte-Carlo simulations with $10^{3}$ samples in each. Estimation results for $\partial {\hat{P}}_{F}/\partial \mu_{i}$ and $\partial {\hat{P}}_{F}/\partial \sigma_{i}$ (i=1,⋯,9) are agreed well with the benchmark result. The Gaussian quadrature scheme was employed to evaluate low-variate integrals for the fractional moment and the moment-based sensitivity analysis. The total number of functional evaluations is 42=1+(5−1)×4+5×5 in this example, which is rather small as compared to that of the brute-force Monte-Carlo simulation based on $10^{5}$ samples.

### 4.2. Reliability-Based Sensitivity Analysis of a Cracked Membrane

A thermal introduced crack is observed in a membrane due to variations of temperature in a heating environment. The membrane is heated with a permanent uniform temperature field $T_{0}$, whereas the temperature is reduced to the ambient temperature *T* during a maintenance procedure. The heat drop implies the tension and the open of a mode I crack. In this regard, the stress intensity factor $K_{\mathrm{IC}}$ of the crack produced by the heat variation can be evaluated as [41]

(42) $$K_{\mathrm{IC}}\left(X\right)=-cE\left(T-T_{0}\right)\sqrt{\frac{\pi a}{\cos(\pi a/4B)}}\left[1-\frac{1}{40}{\left(\frac{a}{2B}\right)}^{2}+\frac{3}{50}{\left(\frac{a}{2B}\right)}^{4}\right]$$

Note that the probability distribution of the stress intensity factor $K_{\mathrm{IC}}\left(X\right)$ is estimated based on the MaxEnt procedure in Equation (19). Therefore, with the material toughness parameter $K_{C}$, the performance function for reliability analysis of the membrane can be defined as

(43) $$g\left(X\right)=K_{C}-K_{\mathrm{IC}}\left(X\right)$$

and the probability of exceedance of the cracked membrane is determined as

$$\mathrm{POE}\left(K_{C}\right)=\mathrm{Pr}\left[K_{\mathrm{IC}}\left(X\right)>K_{C}\right]$$

Herein, the probabilistic characteristic of input random variables are listed in Table 3.

**Figure 5:**
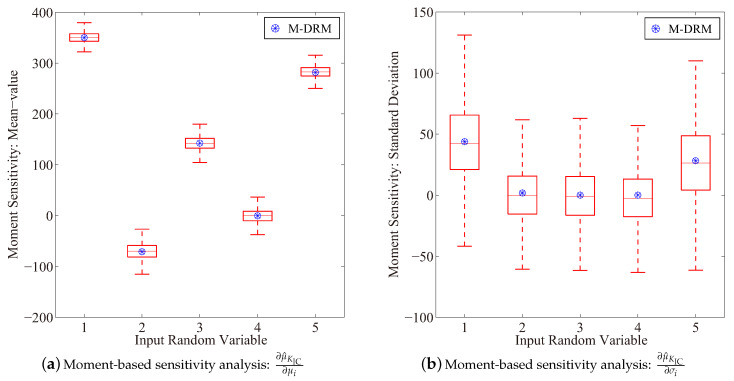
Results of the moment-based sensitivity index (Boxplot: 10^3^ rounds simulation with 10^3^ samples in each).

Figure 5 depicts numerical results for the mean-value based sensitivity index of the cracked membrane structure. Compared to benchmark results for $\partial {\hat{\mu }}_{K_{\mathrm{IC}}}/\partial \mu_{i}$ and $\partial {\hat{\mu }}_{K_{\mathrm{IC}}}/\partial \sigma_{i}$ provided by the brute-force Monte-Carlo simulation, it is observed the high numerical accuracy of this proposed approach.

**Figure 6:**
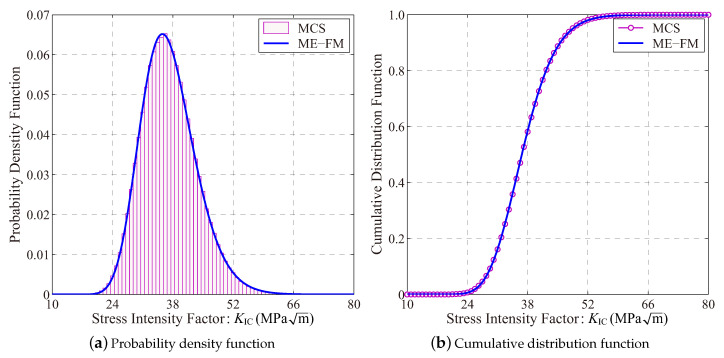
Probability distribution of the stress intensity factor (ME-FM: MaxEnt with fractional moment; MCS: The Monte-Carlo simulation with 10^6^ samples).

With the MaxEnt optimization and the fractional moment approach, the probability distribution for the stress intensity factor $K_{\mathrm{IC}}\left(X\right)$ of the membrane structure is depicted as shown in Figure 6, whereas results for the MaxEnt parameters, i.e., λ and α are listed in Table 4. Compared to the benchmark result provided by the brute-force MCS with $10^{6}$ samples, it has highlighted numerical accuracy of this fractional moment based entropy approach in estimating the probability distribution for the multivariate intensity factor function.

**Figure 7:**
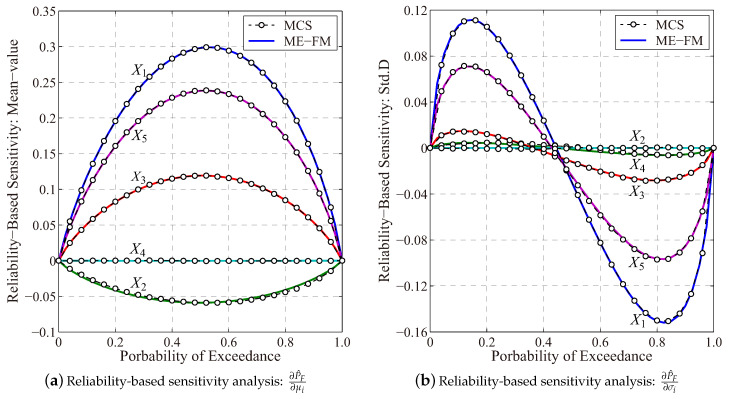
Reliability-based sensitivity analysis of the cracked membrane (ME-FM: The MaxEnt with fractional moment; MCS: Monte-Carlo simulation with 10^6^ samples).

Figure 7 further depicts results for the reliability-based sensitivity analysis of the cracked membrane provided by the proposed entropy approach. The mean-values of input random variables $X_{1}$, $X_{3}$ and $X_{5}$ contribute positively to the structural failure failure, whereas a minimization of the temperature difference, i.e., ($T-T_{0}$) is able to reduce the structural failure probability. Similar observations for the moment-based sensitivity result are presented in Figure 5. Besides, the utility of the multiplicative dimensional reduction method needs 25 functional evaluations in total. This has demonstrated the high numerical efficiency of this approach as well.

### 4.3. Probabilistic Sensitivity Analysis for Fundamental Natural Frequency of a Vehicle Frame

The example considers the probabilistic sensitivity analysis of a vehicle frame that is depicted by a finite element model. The total length (*L*) of the vehicle frame is equally spaced as six segments, i.e., d=L/6. The shell element with the thickness *t* is used to develop the finite element model for natural frequency analysis of the vehicle structure. The simulation model contains 2820 quadratic elements with 15,335 degrees of freedom. The probabilistic characteristic of input random variables are summarized in Table 5, whereas the mean-value of X determines the fundamental natural frequency of the vehicle frame as 31.29 rad/s.

**Figure 8:**
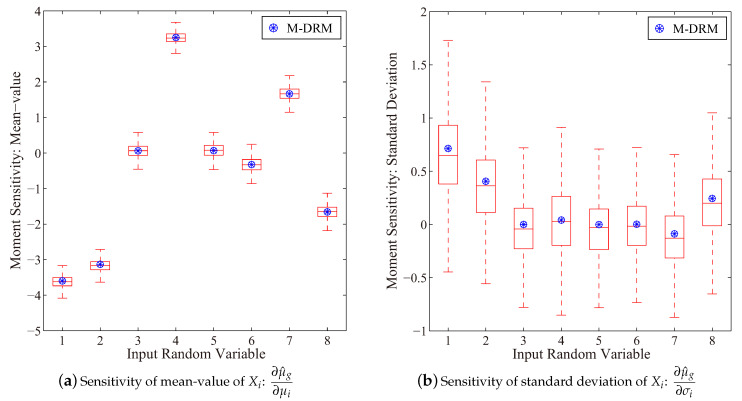
Sensitivity on mean-value of the fundamental frequency (Boxplot: 10^3^ rounds simulation with 10^3^ samples in each).

Figure 8 first presents the sensitivity results for the fundamental natural frequency of the vehicle frame. It has observed that an increase of mean-values $\mu_{4}$ and $\mu_{7}$ is able to increase the mean value response for the fundamental natural frequency of this vehicle frame. However, an increment of parameters $\mu_{1}$ and $\mu_{2}$ will reduce the mean-value natural frequency result. In this regard, distribution parameters, i.e., $\mu_{i}$ and $\sigma_{i}$ of the input random variable can be used to manipulate the response moment results for fundamental natural frequency of the vehicle frame structure.

**Figure 9:**
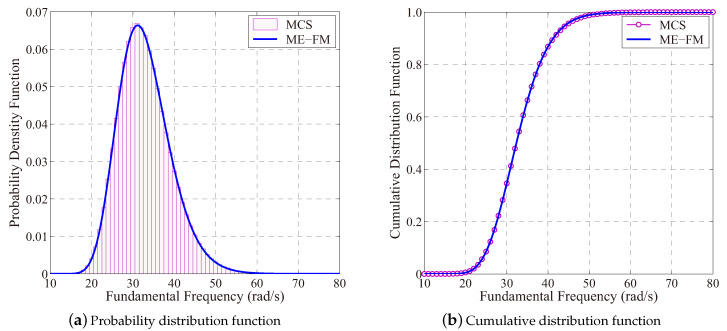
The result of probability distribution of fundamental frequency of the vehicle frame (ME-FM: MaxEnt with fractional moment; MCS: The Monte-Carlo simulation with 10^6^ samples).

To determine the response distribution of the structural natural frequency, the multiplicative dimensional reduction method is first used to determine fractional moments, whereas the MaxEnt optimization procedure is followed to determine an estimation for the probability distribution function for the natural frequency result, and the distribution parameters are summarized in Table 6. Compared to the benchmark result provided by the brute-force Monte-Carlo simulation method, results depicted in Figure 9 have confirmed the high accuracy of this entropy approach in estimating the response distribution of the structural natural frequency.

**Figure 10:**
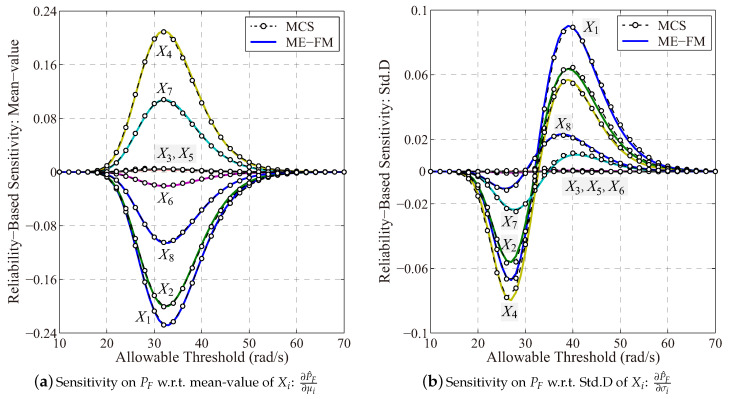
Reliability-based sensitivity analysis on fundamental frequency of the vehicle frame (ME-FM: MaxEnt with fractional moment; MCS: The Monte-Carlo simulation with 10^6^ samples).

Figure 10 presents the reliability-based sensitivity result of the vehicle frame represented by various allowable threshold value of the fundamental natural frequency results. A close agreement between the estimation results of the probability distribution and the sensitivity curves has demonstrate the high accuracy of the proposed approach for probabilistic sensitivity analysis of the vehicle frame. Specially, the mean values of random variables $X_{4}$ and $X_{7}$ increase the natural frequency result positively, whereas $X_{1}$, $X_{2}$, $X_{6}$ and $X_{8}$ change the natural frequency result negatively. Specially, $X_{3}$ and $X_{5}$ almost contribute fairly small for the uncertain natural frequency result, and they can be further treated as deterministic parameters to reduce the dimension of input random variables. Therefore, based on the probability-based sensitivity result, it is possible to locate controlling variables to increase/decrease the structural fundamental natural frequency result to avoid potential failures (e.g., the resonance) of the vehicle frame structure in engineering realities.

## 5. Conclusions

The paper presents a numerical method for the moment-based and the reliability-based sensitivity analysis of a structural model represented by multivariate random variables. In this regard, the structural performance function is first approximated as the product of low-variate component functions, and the utility of the Gaussian quadrature scheme has overcome the curse of dimensionality problem rested in conventional moment calculation procedures. A quasi-analytical expression for the structural response distribution is derived based on the principle of maximum entropy and the fractional moments. There examples are presented to demonstrate potential applications of this approach to rank the significance of input random variables. A rather small number of functional evaluations (≤50 in this paper) were involved in calculating the probability based sensitivity result, which was used to identify controlling variables for response moment and reliability of a multivariate structural model. The sensitivity algorithm was realized based on one-round design of experiment of an investigated structural model. This guarantees numerical efficiency of this approach in reality. Besides, a close agreement of the estimation and the benchmark results has also confirmed its numerical accuracy. To summarize, the principle of maximum entropy and the fractional moment method is able to provide reliable estimation for the reliability-based sensitivity result of a multivariate structural model in general.

## Figures and Tables

**Table 1:** Probabilistic characteristic of random variables for the cantilever tube example.

Variable	Symbol	Unite	Distribution	Mean	COV
*t*	$X_{1}$	mm	Normal	5.0	0.10
*d*	$X_{2}$	mm	Normal	42.0	0.10
$L_{1}$	$X_{3}$	mm	Normal	120.0	0.10
$L_{2}$	$X_{4}$	mm	Normal	50.0	0.10
$F_{1}$	$X_{5}$	kN	Lognormal	3.0	0.10
$F_{2}$	$X_{6}$	kN	Lognormal	3.0	0.10
*P*	$X_{7}$	kN	Lognormal	12.0	0.10
*T*	$X_{8}$	kN	Lognormal	100.0	0.10
$S_{y}$	$X_{9}$	MPa	Lognormal	220.0	0.10

**Table 2:** Parameters for the MaxEnt distribution of g(X).

Entropy	*k*	0	1	2	3
−0.5490	$\lambda_{k}$	−45.85	8.729	78.74	−39.35
	$\alpha_{k}$	−−	−0.8505	0.6785	0.9621
Moment $M_{Y}^{\alpha_{k}}$	MCS	−−	1.7565	0.6615	0.5620
	M-DRM	−−	1.7565	0.6515	0.5621

MCS: The Monte-Carlo simulation with $10^{5}$ samples; M-DRM: Multiplicative dimensional reduction method.

**Table 3:** Random variables of thermal-induced stress intensity factor.

Name	Variable	Symbol	Distribution	Mean	COV
Initial temperature	$T_{0}$	$X_{1}$	Lognormal	$100{\;}^{\circ }C$	0.10
Amphibian temperature	*T*	$X_{2}$	Lognormal	$20{\;}^{\circ }C$	0.10
The size of crack	*a*	$X_{3}$	Lognormal	10 mm	0.10
Width	*B*	$X_{4}$	Normal	200 mm	0.10
Young’s modules	*E*	$X_{5}$	Lognormal	210 GPa	0.10
Thermal expansion Coef.	*c*	–	Deterministic	$12.5\times 10^{-6\;\circ }C^{-1}$	—

**Table 4:** Parameters for the MaxEnt distribution of the stress intensity factor $K_{\mathrm{IC}}\left(X\right)$.

Entropy	*k*	0	1	2	3
3.2455	$\lambda_{k}$	−54.306	1.1317	250.04	128.02
	$\alpha_{k}$	−−	0.8691	−0.6979	−0.6836
Moment $M_{Y}^{\alpha_{k}}$	MCS	−−	23.129	0.0815	0.0858
	M-DRM	−−	23.128	0.0815	0.0858

MCS: The Monte-Carlo simulation with $10^{6}$ samples; M-DRM: Multiplicative dimensional reduction method.

**Table 5:** Probability information of random variables of the vehicle frame structure.

Random Variable		Symbol	Unit	Distribution	Mean	COV
Geometric dimension	*L*	$X_{1}$	mm	Normal	3600	0.10
	*W*	$X_{2}$	mm	Normal	750	0.10
	*w*	$X_{3}$	mm	Normal	90	0.10
	*t*	$X_{4}$	mm	Normal	6	0.10
	*H*	$X_{5}$	mm	Normal	200	0.10
Material property	ν	$X_{6}$	–	Lognormal	0.3	0.10
	*E*	$X_{7}$	MPa	Lognormal	$2.10\times 10^{5}$	0.10
	ρ	$X_{8}$	kg/m${}^{3}$	Lognormal	$7.89\times 10^{3}$	0.10

**Table 6:** Parameters for the MaxEnt distribution of the structural fundamental natural frequency.

Entropy	*k*	0	1	2	3
3.2303	$\lambda_{k}$	−517.8	541.9	163.5	19.1
	$\alpha_{k}$	−−	−0.3063	0.1639	0.2442
Moment $M_{Y}^{\alpha_{k}}$	MCS	−−	0.3453	1.7689	2.3401
	M-DRM	−−	0.3453	1.7689	2.3401

MCS: The Monte-Carlo simulation with $10^{6}$ samples; M-DRM: Multiplicative dimensional reduction method.

## Appendix A. The Kernel Function of Random Variables

### Appendix A.1. The Normal Random Variable

Normal distribution could be represented by the mean-value and its standard deviation as

(A1) $$f_{X}\left(x\right)=\frac{1}{\sqrt{2\pi }\sigma }\exp\left[-{\left(x-\mu \right)}^{2}/\left(2\sigma^{2}\right)\right]$$

Therefore, the kernel function of *X* w.r.t. μ and σ could be directly computed as

(A2) $$\left\{\begin{array}{c} \kappa_{\mu }\left(X\right)=\left(X-\mu \right)/\sigma^{2}\\ \kappa_{\sigma }\left(X\right)=\left(X^{2}-2X\mu +\mu^{2}-\sigma^{2}\right)/\sigma^{3} \end{array}\right.$$

### Appendix A.2. The Lognormal Random Variable

PDF of the Lognormal distribution is

(A3) $$f_{X}\left(x\right)=\frac{1}{\sqrt{2\pi }\;\zeta \;x}\exp\left[-{\left(\log x-\lambda \right)}^{2}/\left(2\zeta^{2}\right)\right]$$

where, the value of λ and ζ could be calculated with the mean-value and standard deviation of *X* as

(A4) $$\left\{\begin{array}{c} \lambda =\log\mu -1/2\log(1+\delta_{X}^{2})\\ \zeta =\sqrt{\log(1+\delta_{X}^{2})} \end{array}\right.$$

where, $\delta_{X}=\sigma /\mu$ is the coefficient of variation (COV). Therefore, with the chain rule of differentiation, the kernel functions are

(A5) $$\left\{\begin{array}{c} \kappa_{\mu }\left(X\right)=\frac{\partial \log\left[f_{X}\left(x\right)\right]}{\partial \lambda }\cdot \frac{\partial \lambda }{\partial \mu }+\frac{\partial \log\left[f_{X}\left(x\right)\right]}{\partial \zeta }\cdot \frac{\partial \zeta }{\partial \mu }\\ \kappa_{\sigma }\left(X\right)=\frac{\partial \log\left[f_{X}\left(x\right)\right]}{\partial \lambda }\cdot \frac{\partial \lambda }{\partial \sigma }+\frac{\partial \log\left[f_{X}\left(x\right)\right]}{\partial \zeta }\cdot \frac{\partial \zeta }{\partial \sigma } \end{array}\right.$$

where,

(A6) $$\left\{\begin{array}{c} \partial \log\left[f_{X}\left(x\right)\right]/\partial \lambda =\left(\lambda -\log X\right)/\zeta^{2}\\ \partial \log\left[f_{X}\left(x\right)\right]/\partial \zeta =\left[\left(\log X-\lambda^{2}\right)-\zeta^{2}\right]/\zeta^{3} \end{array}\right.$$

and,

(A7) $$\left\{\begin{array}{cc} \partial \lambda /\partial \mu =\left(\mu^{2}+2\sigma^{2}\right)/\left[\mu (\mu^{2}+\sigma^{2})\right];\; & \partial \zeta /\partial \mu =\sigma^{2}/\left[\mu \left(\mu^{2}+\sigma^{2}\right)\sqrt{\log(\mu^{2}+\sigma^{2})-2\log\mu }\right]\\ \partial \lambda /\partial \sigma =-\sigma /(\mu^{2}+2\sigma^{2}); & \partial \zeta /\partial \sigma =\sigma /\left[\left(\mu^{2}+\sigma^{2}\right)\sqrt{\log(\mu^{2}+\sigma^{2})-2\log\mu }\right] \end{array}\right.$$

### Appendix A.3. The Weibull Random Variable

Two-parameter Weibull distribution with the pdf of

(A8) $$f_{X}\left(x\right)=a/b{\left(x/b\right)}^{a-1}\exp\left[-{\left(x/b\right)}^{a}\right]\;\left(x\geq 0\right)$$

where, a>0 and b>0 denote the shape and scale parameters, respectively. And the mean-value and standard deviation of *X* are

(A9) $$\left\{\begin{array}{c} \mu =b\Gamma (1+1/a)\\ \sigma =b\sqrt{\Gamma \left(1+2/a\right)-\Gamma^{2}\left(1+1/a\right)} \end{array}\right.$$

Then, the Jacobian matrix of μ and σ in terms of *a* and *b* can be determined as

(A10) $$\left[\begin{array}{cc} \frac{\partial \mu }{\partial a} & \frac{\partial \mu }{\partial b}\\ \frac{\partial \sigma }{\partial a} & \frac{\partial \sigma }{\partial b} \end{array}\right]=\left[\begin{array}{cc} -b\Psi \left(1+1/a\right)\Gamma \left(1+1/a\right)/a^{2} & \Gamma (1+1/a)\\ b^{2}\left[\Psi \left(1+1/a\right)\Gamma^{2}\left(1+1/a\right)-\Psi \left(1+2/a\right)\Gamma \left(1+2/a\right)\right]/\left[a^{2}\sigma \right] & \sigma /b \end{array}\right]$$

in which, Ψ[·] is the digamma function, defined as the logarithmic derivative of Gamma function

(A11) $$\Psi \left(x\right)=d\log\left[\Gamma \left(x\right)\right]/dx=\Gamma^{\prime }\left(x\right)/\Gamma \left(x\right)$$

Therefore, the kernel function of Weibull distribution also could be derived through the chain rule w.r.t. the shape and scale parameters

(A12) $$\left\{\begin{array}{c} \kappa_{\mu }\left(X\right)=\frac{\partial \log\left[f_{X}\left(x\right)\right]}{\partial a}\cdot \frac{\partial a}{\partial \mu }+\frac{\partial \log\left[f_{X}\left(x\right)\right]}{\partial b}\cdot \frac{\partial b}{\partial \mu }\\ \kappa_{\sigma }\left(X\right)=\frac{\partial \log\left[f_{X}\left(x\right)\right]}{\partial a}\cdot \frac{\partial a}{\partial \sigma }+\frac{\partial \log\left[f_{X}\left(x\right)\right]}{\partial b}\cdot \frac{\partial b}{\partial \sigma } \end{array}\right.$$

where,

(A13) $$\left\{\begin{array}{c} \partial \log\left[f_{X}\left(x\right)\right]/\partial a=\left[1+a\log\left(X/b\right)-a\log\left(X/b\right){\left(X/b\right)}^{a}\right]/a\\ \partial \log\left[f_{X}\left(x\right)\right]/\partial b=\left[a{\left(X/b\right)}^{a}-a\right]/b \end{array}\right.$$

and, the derivatives of *a* and *b* w.r.t. μ and σ could be evaluated through the inverse of Jacobian matrix in Equation (A10), i.e.,

(A14) $$\left[\begin{array}{cc} \frac{\partial a}{\partial \mu } & \frac{\partial b}{\partial \mu }\\ \frac{\partial a}{\partial \sigma } & \frac{\partial b}{\partial \sigma } \end{array}\right]={\left[\begin{array}{cc} -b\Psi \left(1+1/a\right)\Gamma \left(1+1/a\right)/a^{2} & \Gamma (1+1/a)\\ b^{2}\left[\Psi \left(1+1/a\right)\Gamma^{2}\left(1+1/a\right)-\Psi \left(1+2/a\right)\Gamma \left(1+2/a\right)\right]/\left[a^{2}\sigma \right] & \sigma /b \end{array}\right]}^{-1}$$
